# Transcranial Direct Current Stimulation (tDCS) Effects on Quantitative Sensory Testing (QST) and Nociceptive Processing in Healthy Subjects: A Systematic Review and Meta-Analysis

**DOI:** 10.3390/brainsci14010009

**Published:** 2023-12-21

**Authors:** Francisco Gurdiel-Álvarez, Yeray González-Zamorano, Sergio Lerma-Lara, Julio Gómez-Soriano, Juan Luis Sánchez-González, Josué Fernández-Carnero, Víctor Navarro-López

**Affiliations:** 1 International Doctorate School, Department of Physical Therapy, Occupational Therapy, Rehabilitation and Physical Medicine, Universidad Rey Juan Carlos, 28933 Alcorcón, Spain; franfisiotmno@gmail.com (F.G.-Á.); yeray.gonzalez@urjc.es (Y.G.-Z.); 2 Cognitive Neuroscience, Pain, and Rehabilitation Research Group (NECODOR), Faculty of Health Sciences, Rey Juan Carlos University, 28032 Madrid, Spain; 3 Department of Physical Therapy, Occupational Therapy, Rehabilitation and Physical Medicine, Rey Juan Carlos University, 28032 Madrid, Spain; victor.navarro@urjc.es; 4 Brain Injury and Movement Disorders Neurorehabilitation Group (GINDAT), Institute of Life Sciences, Francisco de Vitoria University, 28223 Pozuelo de Alarcón, Spain; 5 Department of Physical Therapy, Centro Superior de Estudios Universitarios La Salle, Universidad Autónoma de Madrid, 28023 Madrid, Spain; sergio.lerma@lasallecampus.es; 6 Toledo Physiotherapy Research Group (GIFTO), Faculty of Physiotherapy and Nursing, Universidad Castilla La Mancha, 45071 Toledo, Spain; julio.soriano@uclm.es; 7 Faculty of Nursing and Physiotherapy, Department of Nursing and Physiotherapy, Instituto de Investigación Biomédica de Salamanca (IBSAL), University of Salamanca, Campus Miguel de Unamuno s/n, 37007 Salamanca, Spain; juanluissanchez@usal.es; 8 La Paz Hospital Institute for Health Research, IdiPAZ, 28922 Madrid, Spain; 9 Musculoskeletal Pain and Motor Control Research Group, Faculty of Sport Sciences, Universidad Europea de Madrid, 28670 Madrid, Spain; 10 Movement Analysis, Biomechanics, Ergonomics, and Motor Control Laboratory, Faculty of Health Sciences, Rey Juan Carlos University, 28922 Madrid, Spain

**Keywords:** transcranial direct current stimulation, central sensitization, healthy subjects, pain management

## Abstract

Background: The aim of this study is to determine the effect that different tDCS protocols have on pain processing in healthy people, assessed using quantitative sensory tests (QST) and evoked pain intensity. Methods: We systematically searched in EMBASE, CINAHL, PubMed, PEDro, PsycInfo, and Web of Science. Articles on tDCS on a healthy population and regarding QST, such as pressure pain thresholds (PPT), heat pain thresholds (HPT), cold pain threshold (CPT), or evoked pain intensity were selected. Quality was analyzed using the Cochrane Risk of Bias Tool and PEDro scale. Results: Twenty-six RCTs were included in the qualitative analysis and sixteen in the meta-analysis. There were no significant differences in PPTs between tDCS and sham, but differences were observed when applying tDCS over S1 in PPTs compared to sham. Significant differences in CPTs were observed between tDCS and sham over DLPFC and differences in pain intensity were observed between tDCS and sham over M1. Non-significant effects were found for the effects of tDCS on HPTs. Conclusion: tDCS anodic over S1 stimulation increases PPTs, while a-tDCS over DLPFC affects CPTs. The HPTs with tDCS are worse. Finally, M1 a-tDCS seems to reduce evoked pain intensity in healthy subjects.

## 1. Introduction

In recent decades, direct current stimulation has emerged as a non-invasive neuromodulation technique applied to the scalp to modulate brain excitability and alter brain activity [1]. Notably, transcranial direct current stimulation (tDCS) can produce effects that persist beyond the stimulation period, influencing neuronal function, making it particularly effective in pain treatment [2,3]. It modulates neural circuits related to pain processing and maintains synaptic effects post-intervention [4].

Various studies have assessed tDCS in pain reduction, particularly targeting the primary motor cortex (M1) [5,6,7] or the dorsolateral prefrontal cortex (DLPFC) [8,9]. The stimulation site, alongside parameters like current density and polarity, significantly influences pain effects [10]. For instance, anodic stimulation of M1 modulates the neuromatrix, aiding in chronic pain management and affecting secondary hyperalgesia and conditioned pain modulation [10,11]. This stimulation also alters functional connectivity across several brain regions including M1, the thalamus, the basal ganglia, the amygdala, the cingulate cortex, and the brainstem [12,13,14]. In contrast, anodic stimulation of the prefrontal cortex enhances cognitive and emotional pain aspects, crucial in pain perception control [15,16]. However, cathodal tDCS seems less effective in these regions [12] but shows promising antinociceptive effects and increased pain tolerance when applied to the anterior cingulate cortex (ACC) [17]. Despite significant findings, including increased pain thresholds in various studies [18], the impact of different tDCS protocols on pain thresholds, such as pressure and thermal pain thresholds, remains inconsistently reported [19,20,21,22,23,24,25,26]. This inconsistency fuels ongoing debate about the most effective tDCS protocol for pain modulation.

Different studies have evaluated how healthy people respond to tDCS, but to date, no meta-analysis has been performed regarding the most effective protocol for influencing pain processing. Our study aims to consolidate the current evidence on the efficacy of various tDCS protocols in healthy individuals. By synthesizing data from quantitative sensory tests and nociceptive processing assessments, we hope to clarify the most effective tDCS approaches for pain-processing modulation.

## 2. Materials and Methods

A systematic review of the literature was performed following PRISMA guidelines [27] and the recommendation of the Cochrane Handbook. A comprehensive review of computerized literature databases and searches to find unpublished trials were performed to minimize publication bias.

### 2.1. Strategy of Search and Study Selection

The search was conducted on 16 November 2023 in the EMBASE, CINAHL, PEDro, PsycInfo, Web of Science, and MEDLINE databases. A different combination of terms in relation to tDCS and psychophysical measures or quantitative sensory tests was used in the search strategy. The complete search strategy can be found in Supplementary Material S1.

This meta-analysis protocol was registered in the International Prospective Register of Systematic Reviews (PROSPERO registration no: CRD42023459336).

### 2.2. Eligibility Criteria

We followed the PICOS framework to organize the inclusion criteria. Population (P): studies that included healthy subjects who were more than 18 years of age, without pain in the last 3 months; Intervention (I): studies that use tDCS as a single treatment, independent of the application site; Comparisons (C): sham tDCS; Outcomes (O): primary outcome that included quantitative sensory testing variable, such as PPT, HPT, cold pain threshold (CPT), or evoked pain intensity; and Study design (S): randomized controlled trials (RCTs) published in the Spanish or English languages.

The exclusion criterion was studies that did not include the stimulation parameters. Two authors screened the titles and abstracts of the initially identified studies to determine if they satisfied the selection criteria. Any disagreement was resolved by consensus. Full-text articles were retrieved for the selected titles, and reference lists of the retrieved articles were inspected to identify additional publications. 

### 2.3. Data Extraction

Two independent reviewers analyzed the quality of all selected articles using the same methodology. Disagreements between reviewers were resolved by consensus by including a third reviewer. Inter-evaluator reliability was determined using the “kappa coefficient” (>0.7 means high level of agreement among evaluators, 0.5–0.7 a moderate level of agreement, and <0.5 a low level of agreement). From each study, we extracted the following data to elaborate the characteristics of the table: (1) number of sessions and average age; (2) current parameters (intensity, duration, current density, technique, and electrode location); (3) measurements; and (4) results. The level of agreement between evaluators was moderate.

### 2.4. Methodological Quality Assessment

The Physiotherapy Evidence Database (PEDro) scoring system was used for evaluating the methodological quality of the selected studies [28]. Two authors independently screened the full-text articles to obtain a score using the PEDro scale. The PEDro tool consists of 11 questions with a maximum score of 10. The following criteria were used for rating the methodological quality of a study: from 9 to 10, “excellent”; 6 to 8, “good”; 4 to 5, “fair”; and <4, “poor” [28]. All studies were included in the analysis regardless of study quality. In the event of any discrepancies between the two reviewers, a consensus was attempted to be reached by discussion. If a full consensus could not be reached between the two reviewers after an exhaustive discussion, the opinion of a third reviewer was obtained, and the proceeding majority consensus was taken.

For the qualitative analysis of selected studies, the criteria for classification of evidence were followed for RCTs [29,30]. The evidence was classified into 5 levels based on methodological quality as follows: (1) “Strong”, consistent findings among multiple high-quality randomized controlled trials; (2) “Moderate”, consistent findings among multiple low-quality randomized controlled trials and/or controlled clinical trials and/or one high-quality randomized controlled trial; (3) “Limited”, one low-quality randomized controlled trial and/or controlled clinical trial; (4) “Conflicting”, inconsistent findings among multiple trials (randomized controlled trials and/or controlled clinical trials); or (5) “No evidence from trials”, no randomized controlled trials and/or controlled clinical trials.

### 2.5. Risk of Bias Assessment

Two reviewers independently assessed the risk of bias in the studies. A revised tool to assess the risk of bias in randomized clinical trials (RoB2) [31] was used to assess the risk of bias in randomized trials. The tool is structured into five domains via which bias could be introduced into the outcome. These were identified based on empirical evidence and theoretical considerations. 

### 2.6. Level of Evidence Assessment

The Grading of Recommendations Assessment, Development and Evaluation (GRADE) [32] approach was used by two independent authors to evaluate the quality of evidence. In case of discrepancies, a third author acted. Evidence quality was assessed on a scale of high, moderate, low, or very low, taking into account several factors. These factors included the presence of study limitations (RoB), consistency of results, unexplained heterogeneity, precision of results, the likelihood of publication bias, and the clarity of evidence direction [33]. Very low quality was assigned when all items posed a serious risk or when more than two items carried a very serious risk. A low quality rating was given when two or three items presented a serious risk, or when one or two items exhibited a very serious risk. In cases where two or three items had a serious risk, or one or two items had a very serious risk, the evidence was also classified as low-quality. When only one item presented a serious risk, the quality was deemed moderate. Finally, evidence was considered high-quality when all items displayed no significant concerns.

### 2.7. Data Synthesis

Meta-analysis was performed using ReviewManager statistical software (version 5.4; Cochrane, London, UK). Effects were investigated by calculating standardized mean differences (SMDs) for change scores from baseline to intervention. For this, the sample size, mean difference, and standard deviations (SDs) were extracted. When the study only reported median and first and third quartile values, they were converted to means and SDs [34,35]. When the authors presented only standard errors, these were converted to SDs. If the study did not present the results, the authors were contacted to request them. If results were not available in this way, means and SDs were estimated from graphs (Image J program, version 1.44; National Institute of Health in Bethesda, MA, USA). If none of this was possible, the study was excluded from the quantitative analysis and the information was presented narratively. 

If the study did not report the preintervention–postintervention mean difference in each group, the mean difference was obtained using the pre–postintervention values. In the absence of SD of the difference, we imputed from other data reported in the study: (1) using other measures reported in the study (e.g., confidence intervals and *p* values, following the principles described in Chapter 6.5.2.2 of the Cochrane Handbook) [36]; or, if that was not possible, (2) using the correlation coefficient of the most similar study included (following the principles described in Chapter 6.5.2.8 of the Cochrane Handbook) [36]; or if that was not possible, (3) using a conservative correlation coefficient of 0.5 [37]. This methodology has been performed in other meta-analyses [38].

A meta-analysis was performed for each measurement and for each type of stimulation: anodic or cathodic. Subgroup analyses were performed for stimulation area and intensity. Subgroup analysis by number of sessions was not performed because almost all studies conducted a single session. Meta-analysis was performed using the inverse variance method and a random-effects model with 95% confidence intervals, as it provided more conservative results in the case of heterogeneity between studies, which was expected. *p* values < 0.05 were considered statistically significant. An effect size (SMD) of 0.8 or greater was considered large, an effect size between 0.5 and 0.8 was considered moderate, and an effect size between 0.2 and 0.5 was considered small.

A sensitivity analysis was performed to evaluate the results. For this purpose, the meta-analysis was performed only using studies with low RoB, and then without studies that imputed the SD value of the difference with a correlation coefficient estimated from another study or with the correlation coefficient of 0.5. Sensitivity analysis was performed when the analysis could be performed in at least 5 studies. Study heterogeneity was assessed using the degree of between-study inconsistency (I^2^). The Cochrane Group has established the following interpretation of the I^2^ statistic: a value from 0 to 40% may not be relevant/important heterogeneity, 30 to 60% suggests moderate heterogeneity, 50 to 90% represents substantial heterogeneity, and 75 to 100% represents considerable heterogeneity [39]. Skewness was assessed using funnel plots according to application method (cathodic, anodic) and stimulation site. These analyses were performed only if the subgroups had at least three studies.

## 3. Results

### 3.1. Study Selection

Electronic searches identified 3888 potential studies for review. After elimination of duplicates, a total of 721 studies remained. A total of 670 studies were excluded on the basis of their titles/abstracts, leaving 51 articles for full-text analysis. Another 22 studies were excluded because they studied interventions other than tDCS, did not include QST assessment, or did not include healthy subjects.

**Figure 1:**
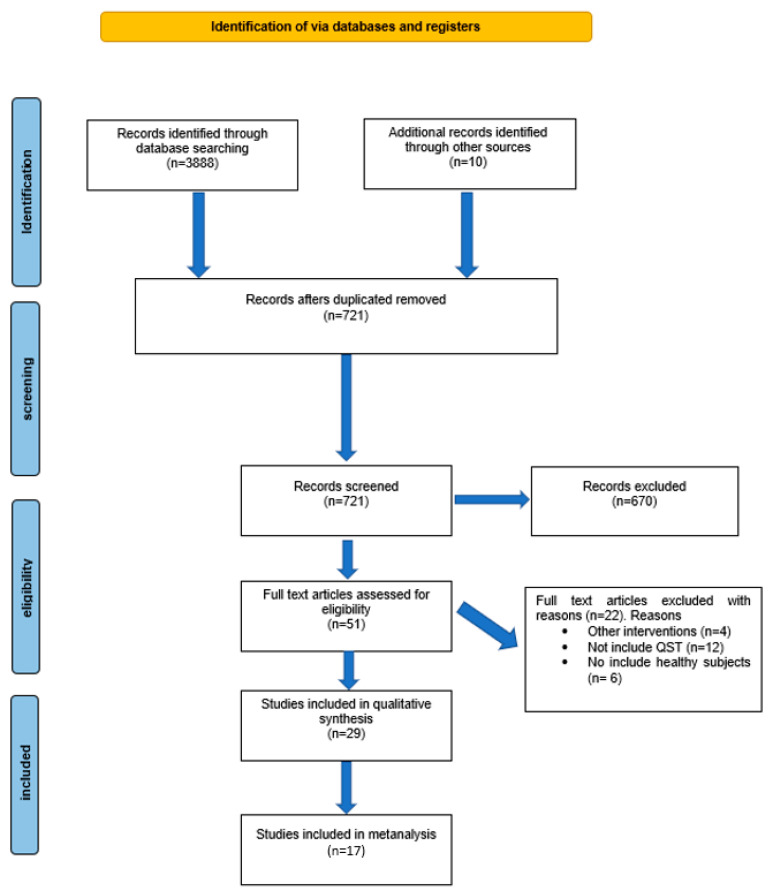
PRISMA flowchart.

Finally, 29 studies [5,9,17,19,20,21,22,23,24,25,26,40,41,42,43,44,45,46,47,48,49,50,51,52,53,54,55,56,57] were included in the qualitative analysis, and 17 [5,9,17,19,20,21,24,26,40,43,44,46,48,50,53,55,56] were included in the quantitative analysis, with a total of 700 subjects. The whole screening process is shown in the PRISMA flow diagram (Figure 1).

### 3.2. Characteristics of the Included Studies

The characteristics of the included studies are shown in Table 1. The average age of the participants was 25.77 years. All subjects included in the studies were healthy and did not present any type of pain at recruitment. All the included studies applied tDCS in some of its modalities, including the study groups that did not apply any therapy in conjunction with tDCS. The agreement between the two reviewers regarding the eligibility and data extraction of the included studies was high according to the kappa coefficient (k = 0.81).

Among the selected studies, most of the studies applied a-tDCS over M1 [5,9,19,21,22,23,24,25,40,42,43,44,47,48,49,50,55,56,57], and other studies applied a-tDCS over DLPFC [9,22,26,43,47,51,52,53,54], S1 [24,53], and SO [19]. Six studies applied c-tDCS over M1 [21,24,40,47,54,57], two over DLPFC [26,47], two over S1 [45,48], and one over ACC [17]. Seven studies applied HD-tDCS [9,17,22,23,42,52,55]. Regarding current density, five applied a current density of 0.028 mA/cm^2^ [21,24,41,45], two applied 0.04 mA/cm^2^ [19,44], nine applied 0.057 mA/cm^2^ [5,25,26,40,46,47,50,57], and one applied 0.1 mA/cm^2^ [53]; the rest of the studies did not report current density. Regarding stimulation time, one applied tDCS for 7 min [38], three for 10 min [23,52], five for 15 min [21,24,41,45,57], eighteen for 20 min [5,9,19,22,26,42,43,44,47,48,49,50,51,53,54,55,56], and one for 40 min [46]. Most studies conducted single treatment sessions [5,17,19,21,23,25,26,40,41,42,43,45,46,47,50,52,55,56,57], and the rest between three and five sessions [9,22,24,44,51,53,54]. 

Regarding outcome measures, 12 measured PPTs [9,17,19,21,22,23,41,44,45,48,52,53,55], 13 measured VAS or NRS [5,21,24,40,43,46,47,49,51,54,56], 10 measured HPTs [5,9,17,21,24,26,40,41,45,48], and 13 measured CPTs [9,17,19,22,25,41,42,43,45,47,48,49,57].

### 3.3. Methodological Quality and Risk of Bias

**Figure 2:**
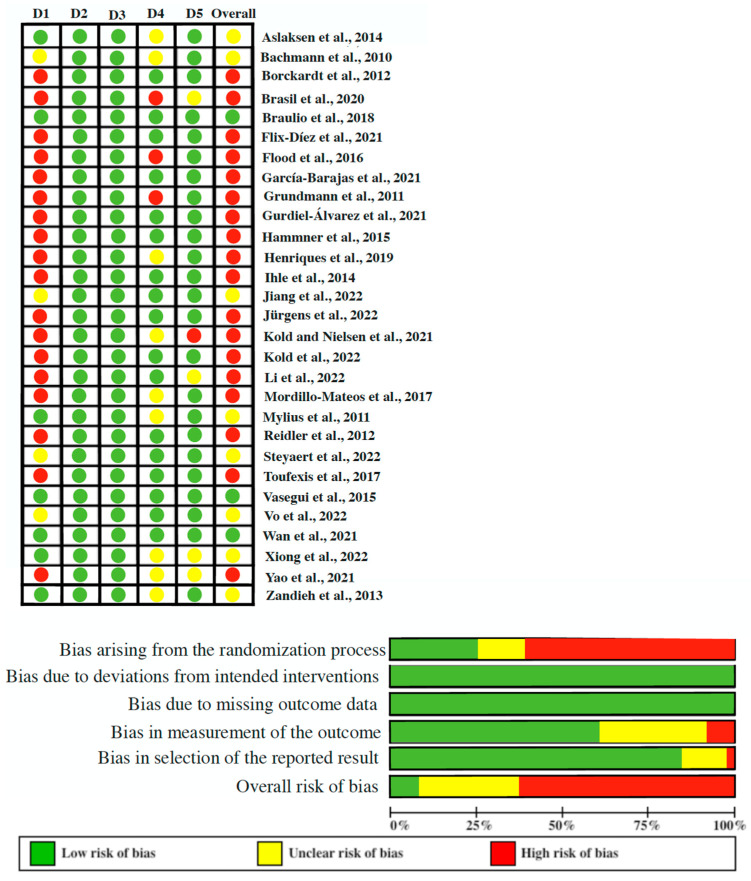
Risk of bias results of the included studies [5,9,17,19,20,21,22,23,24,25,26,40,41,42,43,44,45,46,47,48,49,50,51,52,53,54,55,56,57]. Green color = low risk of bias; Yellow Color= unclear risk of bias and Red color= High risk of bias.

Table 2 shows the details of the PEDro scale and the total score for each of the studies included. The methodological quality score ranged from 5 to 10 out of a maximum of 10 points. The mean methodological quality score of the included studies was 8. Most of the included studies had “good” methodological quality, eight had “excellent” quality, and one had “fair” quality. The most frequent biases were in the randomization process and in the blinding of the assessors (Figure 2). In the reliability analysis, the agreement between the two reviewers regarding the methodological quality of the included studies was high according to the kappa coefficient (k = 0.87).

### 3.4. Certainty of Evidence (GRADE)

Supplementary Material S2 compiles the results of the GRADE assessment, outlining factors such as the risk of bias, inconsistency of results, indirect evidence, imprecision of results, and the likelihood of publication bias. A very serious inconsistency of results (heterogeneity) and risk of bias were downgraded to a very low level of evidence for the overall effect of anodal tDCS for PPTs, VAS, and HPTs. Moreover, a very serious risk of bias led to a reduction in the overall quality of evidence to a low level of anodal tDCS for CPTs, and cathodal tDCS for HPTs and CPTs. Finally, in the context of cathodal tDCS for PPTs, only a serious risk of bias reduced the quality of evidence to a moderate level.

### 3.5. Effects of tDCS on PPTs

**Figure 3:**
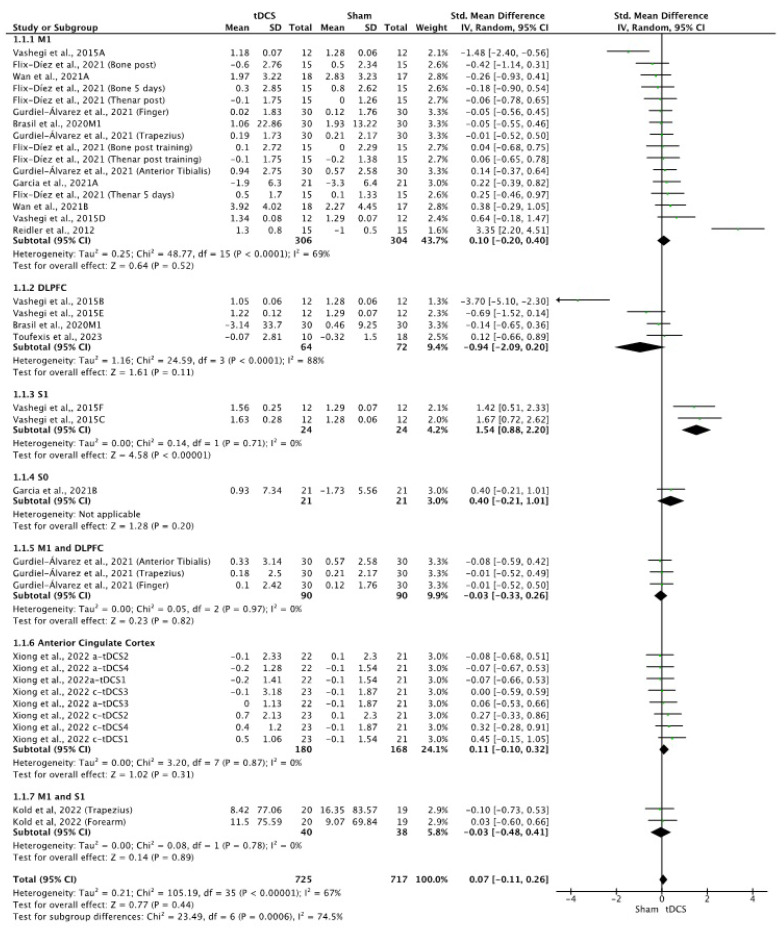
Forest plot of the results of a random-effects meta-analysis shown as SMD, with 95% confidence interval (CI) for the comparison of PPT in the tDCS group and the control group. The shaded square represents the point estimate for each individual study and the weight of the study in the meta-analysis. The diamond represents the overall mean difference of the studies [17,19,20,44,48,53,55].

**Figure 4:**
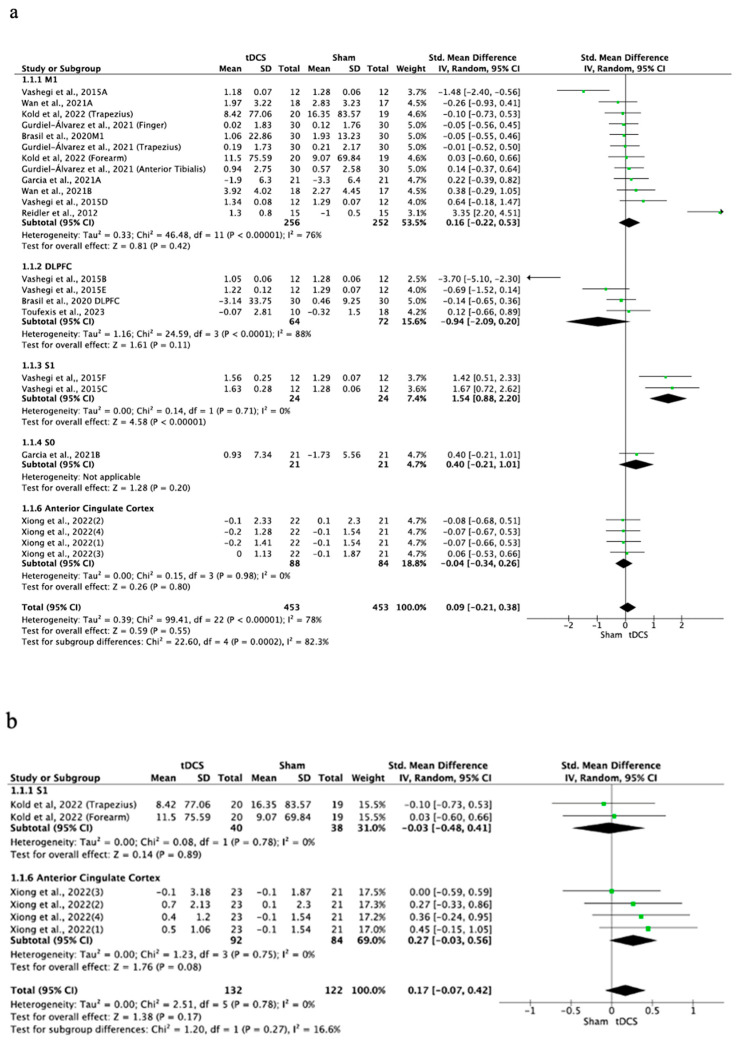
Forest plot of the results of a random-effects meta-analysis shown as SMD, with 95% confidence interval (CI) for the comparison of PPT in the a-tDCS group and the control group (**a**) and in the c-tDCS group and the control group (**b**). The shaded square represents the point estimate for each individual study and the weight of the study in the meta-analysis. The diamond represents the overall mean difference of the studies [17,19,20,44,48,53,55].

The effects of tDCS on PPTs were non-significant when compared with the control group (SMD = 0.07; 95% CI: −0.11 to 0.26; *n* = 1442; Z = 0.77; *p* = 0.44) with moderate to substantial heterogeneity (I^2^ = 67%; *p* < 0.001) (Figure 3). The effects of a-tDCS (SMD = 0.09; 95% CI: −0.21 to 0.38; *n* = 906; Z = 0.59; *p* = 0.55; I^2^ = 78%; *p* < 0.001) and c-tDCS were also non-significant compared to placebo stimulation (SMD = 0.17; 95% CI: −0.07 to 0.42; *n* = 254; Z = 1.38; *p* = 0.17; I^2^ = 0%; *p* = 0.78) (Figure 4). Subgroup analysis showed that there were significant differences between the stimulation sites for the overall tDCS (*p* < 0.001) and a-tDCS (*p* < 0.001) analyses. In both analyses, it was observed that the application of anodic tDCS over S1 produced significant improvements in favor of tDCS compared to placebo (SMD = 1.54; 95% CI: 0.88 to 2.2; *n* = 48; Z = 4.58; *p* < 0.001; I^2^ = 0%; *p* = 0.71). A sensitivity analysis was performed eliminating the study by Reidler et al. [50] as an outlier, without modifying the results of the meta-analysis. In a sensitivity analysis conducted only on studies with a lower RoB, no change in heterogeneity or overall effect size was observed. In the analysis performed by eliminating studies for which the SD was imputed with a correlation coefficient of other studies or for which 0.5 was applied, no differences were observed, the heterogeneity was augmented from I^2^ = 67%; *p* < 0.001 to I^2^ = 90%; *p* < 0.001, and an increase in the overall effect was observed (SMD = 0.07; 95% CI: −0.11 to 0.26; to SMD = 0.21; 95% CI: −0.55 to 0.96). The significance of the application of tDCS on S1 was maintained. The funnel plot showed no asymmetry (Supplementary Material S3).

### 3.6. Effects of tDCS on HPTs

**Figure 5:**
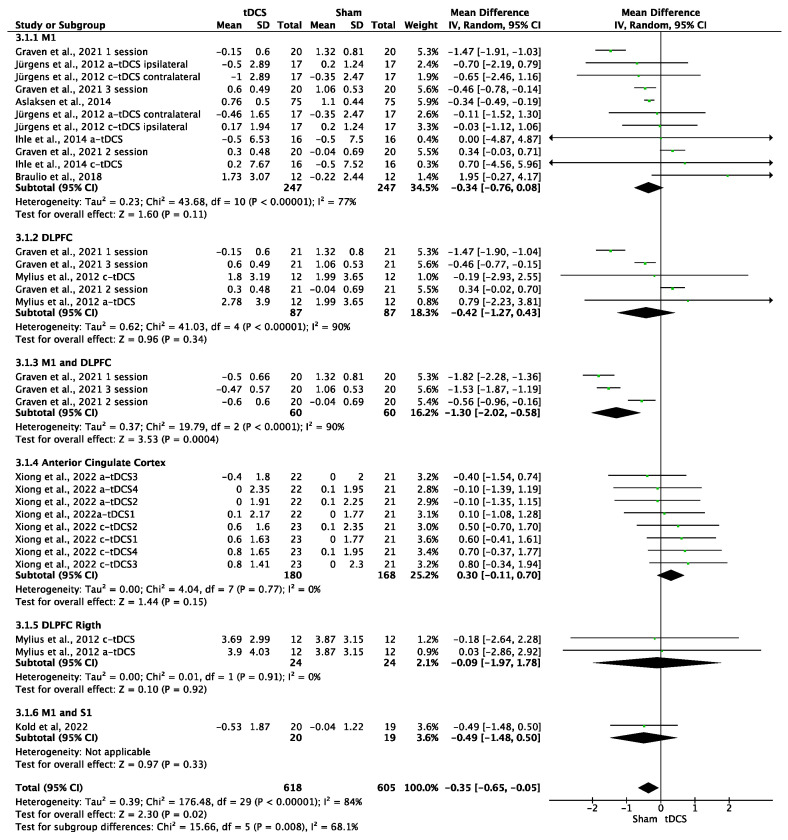
Forest plot of the results of a random-effects meta-analysis shown as MD, with 95% confidence interval (CI) for the comparison of HPT in the tDCS group and the control group. The shaded square represents the point estimate for each individual study and the weight of the study in the meta-analysis. The diamond represents the overall mean difference of the studies [5,9,17,21,24,26,48].

**Figure 6:**
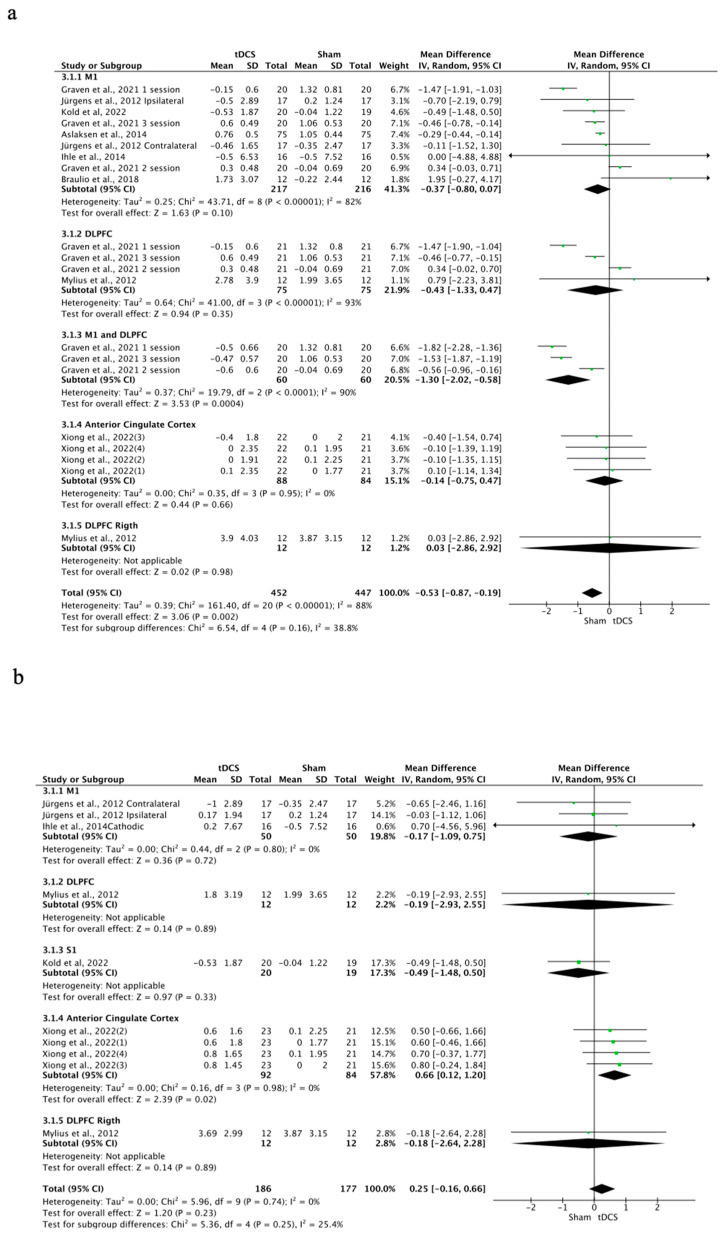
Forest plot of the results of a random-effects meta-analysis shown as MD, with 95% confidence interval (CI) for the comparison of HPT in the a-tDCS group and the control group (**a**) and in the c-tDCS group and the control group (**b**). The shaded square represents the point estimate for each individual study and the weight of the study in the meta-analysis. The diamond represents the overall mean difference of the studies [5,9,17,21,24,26,48].

The effects of tDCS on HPTs were significant in favor of the control group (MD = −0.35; 95% CI: −0.65 to −0.05; *n* = 1223; Z = 2.3; *p* = 0.02) (Figure 5). Heterogeneity was moderate to substantial (I^2^ = 84%; *p* < 0.001). The effects of a-tDCS on HPTs were significant in favor of the control group (MD = −0.53; 95% CI: −0.87 to −0.19; *n* = 899; Z = 3.06; *p* = 0.002; I^2^ = 88%; *p* < 0.001) (Figure 6), but non-significant in the case of c-tDCS compared to sham stimulation (MD = 0.25; 95% CI: −0.16 to 0.66; *n* = 363; Z = 1.2; *p* = 0.23; I^2^ = 0%; *p* = 0.74) (Figure 6). Subgroup analysis showed that there were significant differences between the stimulation sites for the overall tDCS (*p* = 0.008); it was observed that when tDCS was applied simultaneously to M1 and DLPFC, significant improvements occurred in favor of the sham stimulation (MD = −1.3; 95% CI: −2.02 to −0.58; *n* = 120; Z = 3.53; *p* < 0.001; I^2^ = 90%; *p* < 0.001). The sensitivity analysis performed only on studies with a low RoB could not be performed, since only one study included in this analysis showed a low risk of bias [5]. Sensitivity analysis eliminating studies for which the standard deviation (SD) was imputed with a correlation coefficient from other studies and in which a coefficient of 0.5 was used showed no significant differences between real and simulated stimulation (MD = −0.35; 95% CI: −0.92 to −0.21; *n* = 175; Z = 1.22; *p* = 0.22; I^2^ = 0%; *p* = 0.93). The analysis was performed in the studies of Jürgens et al., 2012 [21], and Kold et al., 2022 [48]. The funnel plot showed asymmetry, indicating a possible risk of publication bias (Supplementary Material S4).

### 3.7. Effects of tDCS on CPTs

**Figure 7:**
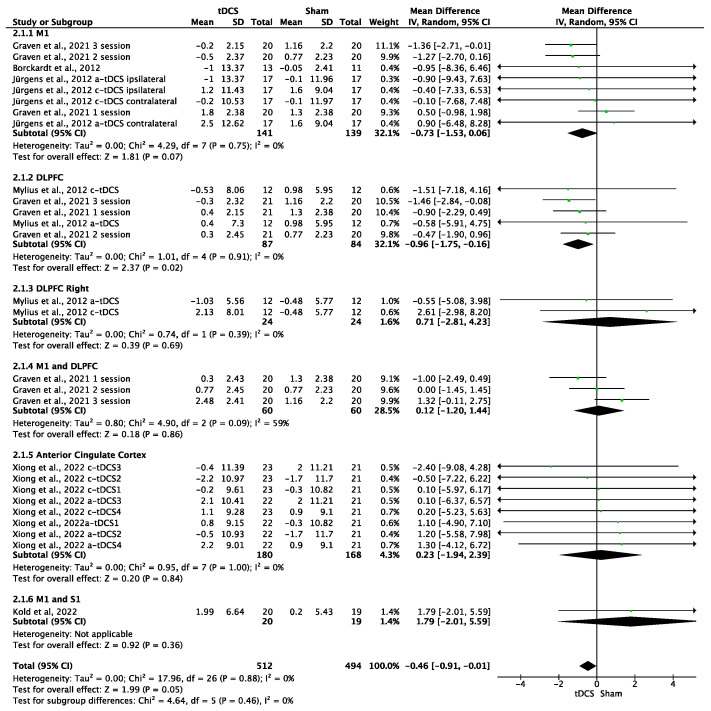
Forest plot of the results of a random-effects meta-analysis shown as MD, with 95% confidence interval (CI) for the comparison of CPT in the tDCS group and the control group. The shaded square represents the point estimate for each individual study and the weight of the study in the meta-analysis. The diamond represents the overall mean difference of the studies [9,17,21,26].

**Figure 8:**
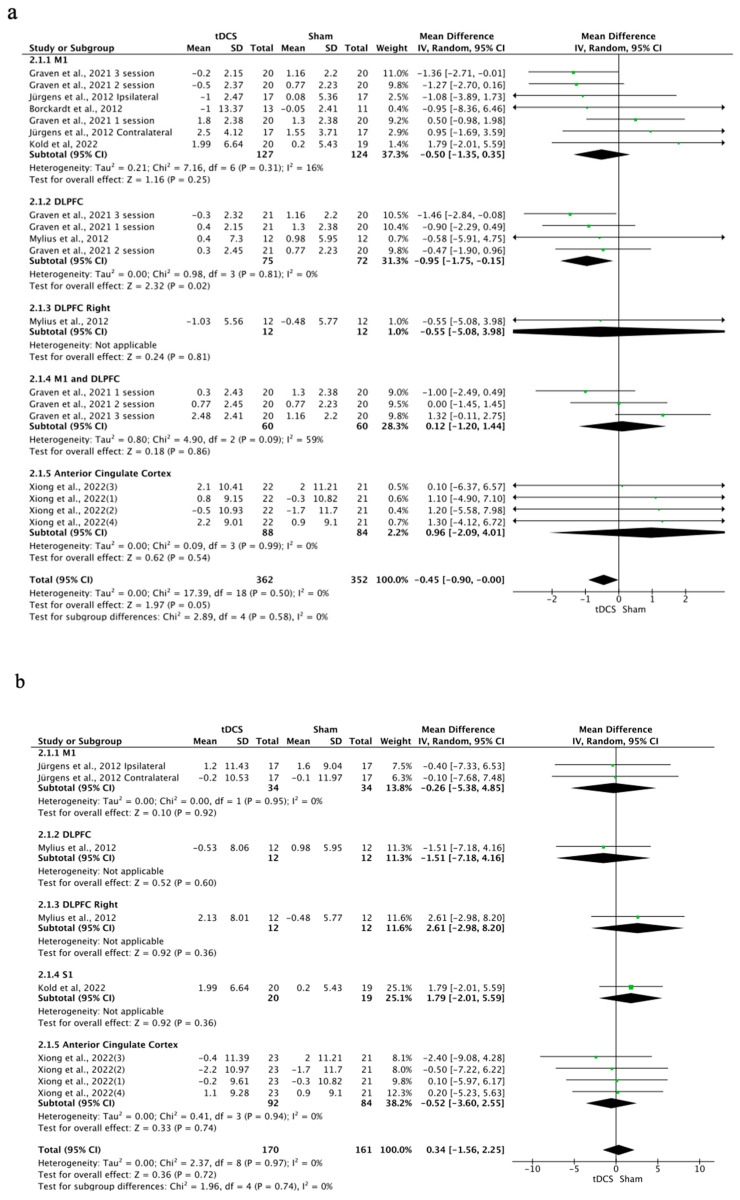
Forest plot of the results of a random-effects meta-analysis shown as MD, with 95% confidence interval (CI) for the comparison of CPT in the a-tDCS group and the control group (**a**) and in the c-tDCS group and the control group (**b**). The shaded square represents the point estimate for each individual study and the weight of the study in the meta-analysis. The diamond represents the overall mean difference of the studies [9,17,21,26,42,48].

The effects of tDCS on CPTs were significant in favor of tDCS compared to sham stimulation (MD = −0.46; 95% CI: −0.91 to −0.01; *n* = 1006; Z = 1.99; *p* = 0.05). Heterogeneity was not relevant (I^2^ = 0%; *p* = 0.88) (Figure 7). The effects of a-tDCS on CPTs were significant in favor of a-tDCS compared to sham tDCS (MD = −0.45; 95% CI: −0.9 to −0.00; *n* = 714; Z = 1.97; *p* = 0.05; I^2^ = 0%; *p* = 0.5) (Figure 8), but non-significant in the case of c-tDCS compared to sham stimulation (MD = 0.34; 95% CI: −1.56 to 2.25; *n* = 331; Z = 0.33; *p* = 0.74; I^2^ = 0%; *p* = 0.97) (Figure 8). Subgroup analysis showed that there were non-significant differences between the stimulation sites (*p* = 0.46), but it was observed that the application of tDCS over DLPFC produced significant improvements in favor of tDCS compared to sham in overall tDCS analyses (MD = −0.96; 95% CI: −1.75 to −0.16; *n* = 171; Z = 2.37; *p* = 0.02; I^2^ = 0%; *p* = 0.91) and a-tDCS analyses (MD = −0.95; 95% CI: −1.75 to −0.15; *n* = 147; Z = 2.32; *p* = 0.02; I^2^ = 0%; *p* = 0.91). The sensitivity analysis performed only on studies with a low RoB could not be performed, as all studies included in this analysis showed a moderate or high risk of bias. Sensitivity analysis eliminating studies for which the standard deviation (SD) was imputed with a correlation coefficient from other studies and in which a coefficient of 0.5 was used showed no significant differences between real and simulated stimulation (MD = 0.85; 95% CI: −1.83 to 3.52; *n* = 175; Z = 0.62; *p* = 0.54; I^2^ = 0%; *p* = 0.96). The analysis was performed in the studies of Jürgens et al., 2012 [21], and Kold et al., 2022 [48]. The funnel plot showed asymmetry, indicating a possible risk of publication bias (Supplementary Material S5).

### 3.8. Effects of tDCS on Pain

**Figure 9:**
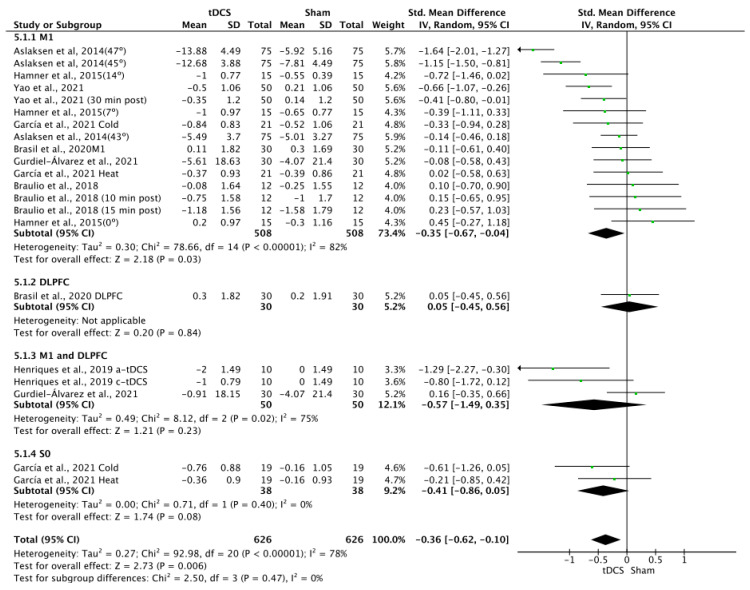
Forest plot of the results of a random-effects meta-analysis shown as SMD, with 95% confidence interval (CI) for the comparison of pain intensity in the tDCS group and the control group. The shaded square represents the point estimate for each individual study and the weight of the study in the meta-analysis. The diamond represents the overall mean difference of the studies [5,19,40,43,46,47,56].

**Figure 10:**
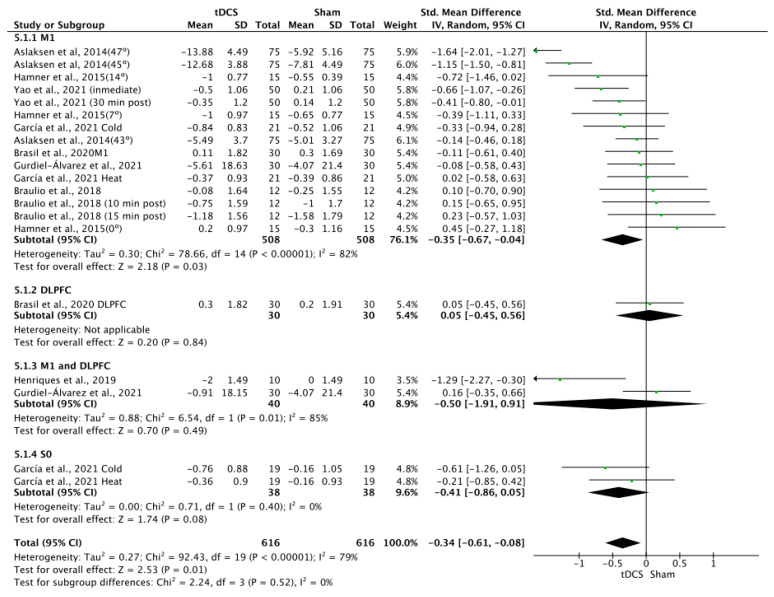
Forest plot of the results of a random-effects meta-analysis shown as SMD, with 95% confidence interval (CI) for the comparison of pain intensity in the a-tDCS group and the control group. The shaded square represents the point estimate for each individual study and the weight of the study in the meta-analysis. The diamond represents the overall mean difference of the studies [5,19,20,40,43,46,56].

The effects of tDCS on pain were significant in favor of tDCS compared to sham stimulation, with a small effect size (SMD = −0.36; 95% CI: −0.62 to −0.1; *n* = 1252; Z = 2.73; *p* = 0.006). Heterogeneity was moderate to substantial (I^2^ = 78%; *p* < 0.001) (Figure 9). The effects of a-tDCS on pain were significant in favor of a-tDCS compared to sham tDCS (SMD = −0.34; 95% CI: −0.61 to −0.08; *n* = 1232; Z = 2.53; *p* = 0.01; I^2^ = 79%; *p* < 0.001) (Figure 10). The effect of c-tDCS on pain was not analyzed due to the lack of studies applying this type of stimulation. Subgroup analysis showed that there were non-significant differences between the stimulation sites, but it was observed that the application of tDCS over M1 produced significant improvements in favor of tDCS compared to sham in overall tDCS (SMD = −0.35; 95% CI: −0.67 to −0.04; *n* = 1016; Z = 2.18; *p* = 0.03; I^2^ = 82%; *p* < 0.001) and a-tDCS analyses (SMD = −0.35; 95% CI: −0.67 to −0.04; *n* = 1016; Z = 2.18; *p* = 0.03; I^2^ = 82%; *p* < 0.001). The sensitivity analysis performed only on studies with a low RoB could not be performed, since only one study included in this analysis showed a low risk of bias [5]. The sensitivity analysis could not be performed by removing studies for which the standard deviation (SD) was imputed with a correlation coefficient from other studies or for which 0.5 was used because all SDs in this group were estimated, except for the study conducted by García et al., 2021 [19]. The funnel plot showed asymmetry, indicating a possible risk of publication bias (Supplementary Material S6).

## 4. Discussion

In this systematic review with meta-analysis, we observed varying effects of tDCS on pain thresholds, as measured by QST, and on pain intensity. For increasing PPTs, only S1 a-tDCS with limited evidence according to the GRADE assessment, was found effective compared to sham stimulation. In the case of CPTs, improvements were supported by low-level evidence for a-tDCS, particularly when applied to DLPFC. However, tDCS appeared less effective than sham for increasing HPTs, backed by very limited evidence. A marginal evidence level also suggests that tDCS might surpass sham stimulation in alleviating evoked pain intensity, especially with M1 stimulation.

Previous meta-analyses identified a modulatory effect of tDCS on increasing pain thresholds, but without differentiating among pain modalities [53,58]. A recent meta-analysis reported reduced evoked pain intensity following tDCS, but no impact on electrical, heat, cold, or pressure pain thresholds [59].

Our meta-analysis showed increased PPTs specifically after a-tDCS over S1, influenced largely by studies from Vaseghi et al. [60]. Notably, only one other study using a lower current density on S1 (0.1 mA/cm^2^ vs. 0.028 mA/cm^2^) found no PPT effects [45], aligning with other research showing no significant difference from sham [59]. While S1’s role in encoding pain location and intensity makes it a plausible target for modulating PPTs [61], more studies are needed to confirm this. 

Regarding CPTs, a-tDCS showed effectiveness over sham stimulation, especially when applied to DLPFC. However, it must be considered that these had moderate to high risk of bias. Comparably, transcranial magnetic stimulation over DLPFC has shown similar CPT effects [62,63]. While a-tDCS has been shown to increase cortical excitability after anode stimulation [1], high-frequency transcranial magnetic stimulation produces a depolarization of neurons on the targeted cortical region and a subsequent increase in excitability [64]. Hence, it could be a parallel effect between the two types of stimulation. Some studies, however, reported no CPT change after a-tDCS or anodic HD-tDCS on DLPFC [22,43]. In these studies, the CPT was measured using cold-water hand immersion and registering time until first sensation of pain. This kind of tonic cold pain stimulus differs from the phasic cold pain stimulus used in the QST, which might explain the variance.

A-tDCS appears to reduce evoked pain intensity in healthy subjects, particularly over M1 as seen in another meta-analysis [60]. Previous studies applying a-tDCS over M1 or M1+DLPFC have found no effect on evoked pain intensity when using phasic mechanical or heat noxious stimulation [21,55]. When using tonic noxious stimulation to evoke pain, a-tDCS over M1 was able to reduce pain intensity without affecting HPTs [40,46]. Nevertheless, in both studies, the effect on pain intensity seemed dependent on the intensity and modality of the noxious stimulus. For noxious heat stimulation, a-tDCS seemed more effective in reducing evoked pain intensity at higher temperatures (47 °C vs. 43–45 °C) [40], whereas for a tonic noxious cold stimulus, the effect was only seen at higher temperatures (14 °C vs. 0–7 °C) [46]. There might be differences in the processing of tonic and phasic noxious stimuli, where tonic stimulation elicits more unpleasantness and tends to have a more emotional processing component than phasic stimulation [65]. Moreover, tDCS over M1 elicits activity changes in the anterior cingulate cortex, basal ganglia, or insula [66,67], which are considered to play a role in affective pain processing [68,69,70,71]. Therefore, the greater effect of a-tDCS in reducing the intensity of pain evoked by noxious tonic stimuli versus noxious phasic stimuli may be due to its effect on these emotional pain processing networks. Future studies should evaluate how tDCS differently affects evoked pain intensity of different modalities and intensities of noxious stimulation.

### Limitations

There are several limitations to be considered in the interpretation of the results. Firstly, data inclusion from all the studies was incomplete due to non-responses from authors. Secondly, although the majority of the included studies were of “good” methodological quality, most of them presented methodological issues, such as inadequate reporting of randomization and blinding protocols. The heterogeneity among studies, especially in the effects on HPTs and pain and the short-term focus of measurements, should be considered, emphasizing the need for longer-term studies. Future research should apply these findings to various pathologies, especially those involving central sensitization processes or chronic pain.

## 5. Conclusions

Our findings suggest that, depending on the stimulation´s polarity and site, tDCS can modulate PPTs, CPTs, and evoked pain intensity by tonic nociceptive stimuli in healthy subjects. There is very limited evidence for the effects of S1 a-tDCS on PPTs and M1 a-tDCS on evoked pain intensity. DLPFC a-tDCS shows low-level evidence in influencing CPTs. Lastly, there is minimal evidence that M1 or M1+DLPFC a-tDCS are less effective than sham stimulation in increasing HPTs.

## Figures and Tables

**Table 1:** Data of included studies.

Author, Year, Design, N	Sessions, Age (Years)	Current Parameters (Intensity, Duration, Current Density, Technique, and Location)	Measurements	Results
Aslaksen et al., 2014, Double-blinded RCT, N = 75 [40]	1 session, Age: 23.5	I = 2 mA, D = 7 min, CD = 0.057 mA/cm^2^, a-tDCS over M1	VAS, HPT	a-tDCS reduced pain intensity but did not increase the HPT.
Bachmann. C, 2010, Single-blinded RCT, N = 8 [41]	1 session, Age: 32.8	I = 1 mA, D = 15 min, CD = 0.028 mA/cm^2^, c-tDCS over M1	PPT, HPT, CPT, CDT, MDT, MPT	c-tDCS significantly increased CDT, mechanical detection threshold (MDT), and mechanical pain threshold (MPT).
Borckardt et al., 2012, Single-blinded RCT, N = 24 [42]	1 session, Age: 26.5	I = 2 mA, D = 20 min, CD = not specified, anodic HD-tDCS 4 × 1 over M1	CDT, CPT, WDT, HPT, MPT, TSP	a-TDCS significantly decreased WDT and CDT, reduced TSP, and marginally increased CPT.
Brasil et al., 2020, Single-blinded RCT N = 30 [43]	1 session, Age: 18.5	I = 2 mA; D = 20 min; CD = 0.057 mA/cm^2^, a-tDCS over M1, a-tDCS over DLPFC	CPT, VAS	tDCS over M1 or DLPFC shows no difference in analgesic effects, pain resistance, and pain tolerance compared to sham tDCS.
Braulio et al., 2018, Double-blinded RCT, N = 48 [5]	1 session, Age: 26.4	I = 2 mA, D = 20 min, CD = 0.057 mA/cm^2^, a-tDCS over M1	NRS, CPM, HPT, TSP	The application of a-tDCS on M1 blocked the disconnection of the descending pain modulatory system.
Flix et al., 2021, Triple-blinded RCT N = 28 [44]	5 sessions, Age: 18–35	I = 1 mA, D = 20 min, CD = 0.04 mA/cm^2^, a-tDCS over M1	PPT, MDT	tDCS over M1 did not produce changes in the somatosensory variables assessed.
Flood et al., 2016, Double-blinded RCT, N = 30 [23]	1 session, Age: 23.9 (4.56)	I = 2 mA, D = 10 min, CD = not specified, anodic HD-tDCS 4 × 1 over M1	PPT, CPM	a-tDCS reduced the pain perceived in the CPM protocol and increased the PPT.
García-Barajas et al., 2021, Double-blinded RCT, N = 40 [19]	1 session, Age: 23.4 (3,6)	I = 1.5 mA; D = 20 min; CD = 0.042 mA/cm^2^; a-tDCS over M1, a-tDCS over S0	PPT, CPT, CPM, HPT	tDCS over M1 or S0 reduced thermal pain intensity to cold, but not to heat or mechanical pain. tDCS showed no effect on CPM.
Grundmann et al., 2011, Single-blinded RCT, N = 12 [45]	1 session, Age: 30	I = 1 mA, D = 15 min, CD = 0.028 mA/cm^2^, a-tDCS, or c-tDCS over S1	CPT, CDT, HPT, PPT	c-tDCS over S1 increased CDT, but not a-tDCS or sham tDCS.
Gurdiel-Álvarez et al., 2021, Triple-blind, crossover N = 30 [20]	1 session, Age: 21.9	I = 2 mA; D = 20 min; CD = 0.1 mA/cm^2^, a-TDCS M1+DLPFC or a-TDCS M1	UDP, CPM, TSP, cold pain intensity	Neither a-TDCS over M1+DLPFC nor a-TDCS over M1 succeed in modulating UDP, CPM, TSP, or cold pain intensity.
Hamner et al., 2015, Single-blinded RCT, N = 15 [46]	1 session, Age: 25.5	I = 2 mA, D = 40 min, CD = 0.057 mA/cm^2^, a-tDCS over M1	VAS	a-tDCS reduced pain intensity (cold pressor test) compared to sham.
Henriques et al., 2019, Single-blinded RCT, N = 10 [47]	1 session, Age: 24 (4).	I = 2 mA, D = 20 min, CD = 0.057 mA/cm^2^, c-tDCS M1 + a-tDCS DLPFC, or c-tDCS DLPFC + a-tDCS M1 bilateral	VAS	c-tDCS M1 + a-tDCS DLPFC reduced pain intensity, but not a-tDCS M1 + c-tDCS DLPFC or sham tDCS.
Ihle et al., 2014, Double-blinded RCT, N = 16 [24]	3 sessions, Age: 27	I = 1 mA, D = 15 min, CD = 0.028 mA/cm^2^, c-tDCS or a-tDCS over M1	NRS, HPT, TSP	Neither c-TDCS nor a-tDCS on M1 significantly modified nociceptive processing nor decreased pain intensity compared to sham.
Jiang et al., 2022, Double-blinded RCT, N = 26 [22]	3 sessions, Age: 22.38	I = 2 mA; D = 20 min; CD = /, anodic HD-tDCS 4 × 1 over M1 or over DLPFC	CPT, CPM, PPT	HD-tDCS over M1 improves CPM, while over DLPFC it had no significant effect.
Jürgens et al., 2012, Single-blinded RCT, N = 17 [21]	1 session, Age: 24.9	I = 1 mA, D = 15 min, CD = 0.028 mA/cm^2^, a-tDCS or c-tDCS over M1	VAS, PPT, HPT, TS	a-tDCS and c-tDCS on M1 did not increase PPT and HPT and did not reduce perceived pain intensity in VAS.
Kold and Nielsenl, 2021, Double-blinded RCT, N = 81 [9]	3 sessions, Age: 25.1 (5.6)	I = 2 mA; D = 20 min; CD = /, anodic HD-tDCS 4 × 1 tDCS over M1, DLPFC or M1+DLPFC	PPT, CPM HPT, CPT	HD-tDCS has not been shown to have an effect on the modulation of somatosensory sensitivity and pain over sham in either M1 or DLPFC.
Kold et al., 2022, Double-blinded RCT N = 20 [48]	1 session, Age: 21.9	I = 2 mA; D = 20 min; CD = /, a-tDCS over M1 and c-tDCS over S1	PPT, HPT, CPT	No effect on PPT, CPT, or HPT on the neck was seen after M1-S1 tDCS compared to sham.
Li et al., 2022, Double-blinded RCT, N = 28 [49]	1 session, Age: 22.92	I = 1 mA; D = 20 min; CD = /, anodic or cathodic HD-tDCS 4 × 1 tDCS over M1	CPT, NRS	Only anodal HD-tDCS significantly increased the cold pain threshold when compared with sham stimulation. Neither anodal nor cathodal HD-tDCS showed significant analgesic effects on CPT or pain intensity.
Mordillo-Mateos et al., 2017, Double-blinded RCT, N = 20 [25]	1 session, Age: 31.9	I = 2/1 mA, D = 15/5 min, CD = 0.057 mA/cm^2^, a-tDCS over M1	CPT	a-tDCS on M1 could modify CPT, but no effect on pain thresholds was observed.
Mylius et al., 2012, Single-blinded RCT, N = 24 [26]	1 session, Age: 22.7	I = 2 mA, D = 20 min, CD = 0.057 mA/cm^2^, a-tDCS or c-tDCS over DLPFC	HPT	a-tDCS over DLPFC increased HPT, but not c-tDCS or sham tDCS.
Reidler et al., 2012, Double-blinded RCT, N = 15 [50]	1 session, Age: 36.7	I = 2 mA, D = 20 min, CD = 0.057 mA/cm^2^, a-tDCS over M1	CPM	a-TDCS over M1 reduced pain intensity and increased UDP in the CPM protocol.
Steyaert et al., 2022 crossover, double-blinded study, N = 19 [51]	3 sessions, Age: 23.5 (4)	I = 2 mA, D = 20 min, CD =/; multichannel a-tDCS over left DLPFC	NRS	a-tDCS over DLPFC modulates the size of the HFS-induced area of secondary mechanical hyperalgesia but does not reduce pain intensity.
Toufexis et al., 2023, Single-blinded RCT, N = 40 [52]	1 session, Age: 22.2	I: 2 mA; D = 10 min; CD = /, anodal HD-tDCS over DLPFC	CPM, PPT	tDCS produced a significant increase in pain modulation capacity. No significant changes were observed in pain sensitivity and stress-induced hyperalgesia.
Vaseghi B, 2015, Double-blinded RCT, N = 12 [53]	4 sessions, Age: 23.6	I = 0.3 mA, D = 20 min, CD = 0.1 mA/cm^2^, a-tDCS over M1, a-tDCS over DLPFC, a-tDCS over S1.	PPT	tDCS anodal stimulation did not increase the PPTs for any group.
Vo et al., 2022, Double-blinded RCT, N = 39 [54]	4 sessions, Age: 26.87 (9.26)	I = 1 mA; D = 20 min; CD = /; a-tDCS over M1, a-tDCS over DLPFC, a-tDCS over M1+DLPFC,	VAS	tDCS on M1 increased moderate evoked pain threshold, tDCS on DLPFC eliminated secondary hyperalgesia. Their combined application does not produce better results.
Wan et al., 2021, Single-blinded RCT, N = 35 [55]	1 session, Age: 23.5 (2.28)	I = 2 mA; D = 20 min; CD = /; anodic HD-tDCS over M1	CPM, PPT	HD-tDCS on M1 improved the analgesic efficacy of CPM in healthy subjects.
Xiong et al., 2022, Double-blinded RCT, N = 66 [17]	1 session, Age: 20.5 (2.4)	I = 2 mA; D = 20 min; CD = /; anodic or cathodic HD-tDCS over Anterior Cingulate Cortex	PPT, HPT, CPT	Cathodic HD-tDCS over the anterior cingulate cortex increased PPT compared to sham tDCS.
Yao et al., 2021, Double-blinded RCT, N = 150 [56]	1 session, Age: 19.82 (0.13)	I = 1 mA; D = 20 min; CD = 0.04 mA/cm^2^; a-tDCS over M1	NRS	a-tDCS immediately reduced pain sensation, and this effect was more pronounced when pain expectation was uncertain.
Zandieh. A, 2013, Single-blinded RCT, N = 22 [57]	1 session, Age: 27.9	I = 2 mA, D = 15 min, CD = 0.057 mA/cm^2^, a-tDCS or c-tDCS over M1	CPT, Time latencies to pain threshold and tolerance	a-tDCS over M1 increased the CPT, but not c-tDCS or sham tDCS. a-tDCS increases the time latencies for threshold cold and pain tolerance, in contrast to cathodic stimulation. tDCS does not alter subjective pain tolerance scores.

a-tDCS: anodic transcranial direct current stimulation; c-tDCS: cathodic transcranial direct current stimulation; CD: current density; CDT: cold detection threshold; CPM: conditioned pain modulation; CPT: cold pain threshold; DLPFC: dorsolateral prefrontal cortex; HD-tDCS: high-definition transcranial direct current stimulation; HPT: heat pain threshold; M1: primary motor cortex; mA: milliampere; MDT: mechanical detection threshold; MPT: mechanical pain threshold; NRS: numerical rating scale; PPT: pressure pain threshold; RCT: randomized clinical trial; S1: primary somatosensory cortex; tDCS: transcranial direct current stimulation; TSP: temporal summation of pain; VAS: visual analogue scale.

**Table 2:** PEDro scale scores for the selected studies.

Author, Year	1	2	3	4	5	6	7	8	9	10	11	Total
Aslaksen et al., 2014 [40]	X	X	X	X	X	X		X	X	X	X	9
Bachmann et al., 2010 [41]	X	X	X	X	X			X	X	X	X	8
Borckardt et al., 2012 [42]	X	X	X		X			X	X	X	X	7
Brasil et al., 2020 [43]	X	X						X	X	X	X	5
Braulio et al., 2018 [5]	X	X	X	X	X	X	X	X	X	X	X	10
Flix-Díez et al., 2021 [44]	X	X	X		X	X	X	X	X	X	X	9
Flood et al., 2016 [23]	X	X	X	X	X			X	X	X	X	8
García-Barajas et al., 2021 [19]	X			X	X	X	X	X	X	X	X	8
Grundmann et al., 2011 [45]	X	X	X	X	X			X	X	X	X	8
Gurdiel-Álvarez et al., 2021 [20]	X	X		X	X	X	X	X		X	X	8
Hamner et al., 2015 [46]	X	X	X	X	X			X	X	X	X	8
Henriques et al., 2019 [47]	X	X	X	X	X			X	X	X	X	8
Ihle et al., 2014 [24]	X	X	X	X	X	X		X	X	X	X	9
Jiang et al., 2022 [22]	X	X		X	X	X		X	X	X	X	8
Jürgens et al., 2012 [21]	X	X	X	X	X			X	X	X	X	8
Kold and Nielsen, 2021 [9]	X	X		X	X	X		X		X	X	7
Kold et al., 2022 [48]	X	X		X	X		X	X	X	X	X	9
Li et al., 2022 [49]		X		X	X		X	X	X	X	X	8
Mordillo-Mateos et al., 2017 [25]	X		X	X	X		X	X	X	X	X	8
Mylius et al., 2011 [26]	X	X	X	X	X			X	X	X	X	8
Reidler et al., 2012 [50]	X	X	X	X	X	X		X	X	X	X	9
Steyaert et al., 2022 [51]	X	X	X	X	X	X	X	X		X	X	9
Toufexis et al., 2023 [52]	X	X	X	X	X			X	X	X	X	8
Vaseghi et al., 2015 [53]	X	X	X	X	X	X	X	X	X	X	X	10
Vo et al., 2022 [54]	X	X		X	X		X	X		X	X	7
Wan et al., 2021 [55]	X	X	X	X	X		X	X	X	X	X	9
Xiong et al., 2022 [17]	X	X	X	X	X		X	X		X	X	8
Yao et al., 2021 [56]	X			X	X	X	X	X		X	X	7
Zandieh et al., 2013 [57]	X	X	X	X	X			X	X	X	X	8

## Data Availability

The data are not publicly available due to the fact that all of it was extracted from available and published works which can be openly found in the literature.

## Supplementary Materials

The following supporting information can be downloaded at: https://www.mdpi.com/article/10.3390/brainsci14010009/s1, S1: Search Strategy; Table S1: GRADE; Figure S2: Funnel plot of tDCS vs. sham tDCS studies evaluating PPT; Figure S3: Funnel plot of tDCS vs. sham tDCS studies evaluating HPT; Figure S4: Funnel plot of tDCS vs. sham tDCS studies evaluating CPT; Figure S5: Funnel plot of tDCS vs. sham tDCS studies evaluating pain intensity.
