# Supplementary material for: Transcranial Direct Current Stimulation (tDCS) Effects on Quantitative Sensory Testing (QST) and Nociceptive Processing in Healthy Subjects: A Systematic Review and Meta-Analysis
Source: Brain Sci. 2023 Dec 21;14(1):9. doi: 10.3390/brainsci14010009 (PMC10813344; doi:10.3390/brainsci14010009)
Supplement: Supplementary file 1 [file brainsci-14-00009-s001.zip › brainsci-2706448-supplementary.pdf]

Supplementary material.

S1. Complete search strategy.

MEDLINE, accessed by PubMed (1990 to march 2023), EMBASE (1990 to march 2023), CINAHL (1990 to march 2023), Web of Science (1990 to march 2023), PsycInfo (1990 to march 2023), and PEDro electronic databases were searched using the medical subject headings (MeSH): "transcranial direct current stimulation", “direct current stimulation”, "pain", "healthy volunteers", "pain measurement", “endogenous pain modulation”, "nociception", "humans", “pain modulation”, “quantitative sensory testing” as well as following free terms, combined in multiple search strategies with Boolean operators in order to find relevant articles: “direct current stimulation”, “sensory testing”, “participants”, “pain measurement”, “clinical trials”, “humans”, “antinociception”, “clinical study”.

A comprehensive generic search string used for each database are included with the boolean operators.

MESH terms used for pubmed, EMBASE and Web Of Science: (("transcranial direct current stimulation"[MeSH Terms] OR ("transcranial"[All Fields] AND "direct"[All Fields] AND "current"[All Fields] AND "stimulation"[All Fields]) OR "transcranial direct current stimulation"[All Fields]) AND ("Current"[Journal] OR "current"[All Fields]) AND stimulation[All Fields]) OR (transcutaneous[All Fields] AND direct[All Fields] AND ("Current"[Journal] OR "current"[All Fields]) AND stimulation[All Fields])) AND (("pain"[MeSH Terms] OR "pain"[All Fields]) OR (quantitative[All Fields] AND sensory[All Fields] AND ("research design"[MeSH Terms] OR ("research"[All Fields] AND "design"[All Fields]) OR "research design"[All Fields] OR "test"[All Fields])) OR ("healthy volunteers"[MeSH Terms] OR ("healthy"[All Fields] AND "volunteers"[All Fields]) OR "healthy volunteers"[All Fields] OR ("healthy"[All Fields] AND "subjects"[All Fields]) OR "healthy subjects"[All Fields]) OR ("healthy volunteers"[MeSH Terms] OR ("healthy"[All Fields] AND "volunteers"[All Fields]) OR "healthy volunteers"[All Fields] OR

("healthy"[All Fields] AND "participants"[All Fields]) OR "healthy participants"[All Fields]) OR ("pain measurement"[MeSH Terms] OR ("pain"[All Fields] AND "measurement"[All Fields]) OR "pain measurement"[All Fields] OR ("pain"[All Fields] AND "measurements"[All Fields]) OR "pain measurements"[All Fields]) OR (("pain"[MeSH Terms] OR "pain"[All Fields]) AND modulation[All Fields]) OR (("pain"[MeSH Terms] OR "pain"[All Fields]) AND modulation[All Fields] AND system[All Fields]) OR (endogenous[All Fields] AND ("pain"[MeSH Terms] OR"pain"[All Fields]) AND modulation[All Fields]).

Terms used for Cinhal

tDCS and Healthy subjects and pain

Terms used for web of PEDro

tDCS and healthy subjects and pain

Supplementary material S2. Provides details of the GRADE assessment.
Table S1.

Number of studies	Risk of Bias	Inconsistency†	Indirectness‡	Imprecision§	Publication Bias¶	MD or SMD (95% CI)	Quality of Evidence
Effects of tDCS on PPTs							
Overall anodal tDCS							
Eight trials (n=906)	Very serious	Serious (I^2 = 78%)	No serious	No serious	No serious	SMD = 0.9 (-0.21, 0.38)	Low
Overall cathodal tDCS							
Two trial (n=235)	Serious	No serious (I^2 = 0%)	No serious	No serious	No serious	SMD = 0.17 (-0.07, 0.42)	Moderate
Effects of tDCS on VAS							
Overall anodal tDCS							
Nine trials (n =1252)	Very serious	Serious (I^2 = 78%)	No serious	No serious	No serious	SMD = -0.36 (-0.62, -0.10)	Very low
Effects of tDCS on HPT							
Overall anodal tDCS							
Eight trials (n=899)	Very serious	Very serious (I^2 = 88%)	No serious	No serious	No serious	SMD = -0.53 (-0.87, -0.19)	Very low
Overall cathodal tDCS							
Five trials (n= 363)	Very serious	No Serious (I^2 = 0%)	No serious	No serious	No serious	SMD = 0.25 (-0.16, 0.66)	Low
Effects of tDCS on CPT							
Overall anodal tDCS							
Five trials (n=714)	Very serious	No serious (I^2 = 0%)	No serious	No serious	No serious	SMD = -0.45 (-0.90, -0.00)	Low
Overall cathodal tDCS							
Three trials (n= 331)	Very serious	No serious (I^2 = 0%)	No serious	No serious	No serious	SMD = 0.34 (-1.56, 2.25)	Low

GRADE, Grading of Recommendations Assessment, Development and Evaluation; MD, mean difference; SMD, standardized mean difference.

*"No," most information is from results at low risk of bias; "serious," crucial limitation for one criterion or some limitations for multiple criteria sufficient to lower confidence in the estimate of effect; "very serious," crucial limitation for one or more criteria sufficient to substantially lower confidence in the estimate of effect.

† "Serious" = $I^2 > 40\%$; "Very serious" = $I^2 > 80\%$.

‡ No indirectness of evidence was found in any study

§ Based on sample size. "Serious," n < 250 subjects; "very serious," n < 250 and the estimated effect is little or absent.

¶ Based on funnel plots. No publication bias was found. Funnel plots are not shown because the number of trials was less than 10.

S3. Effects of tDCS on PPTs funnel plot.

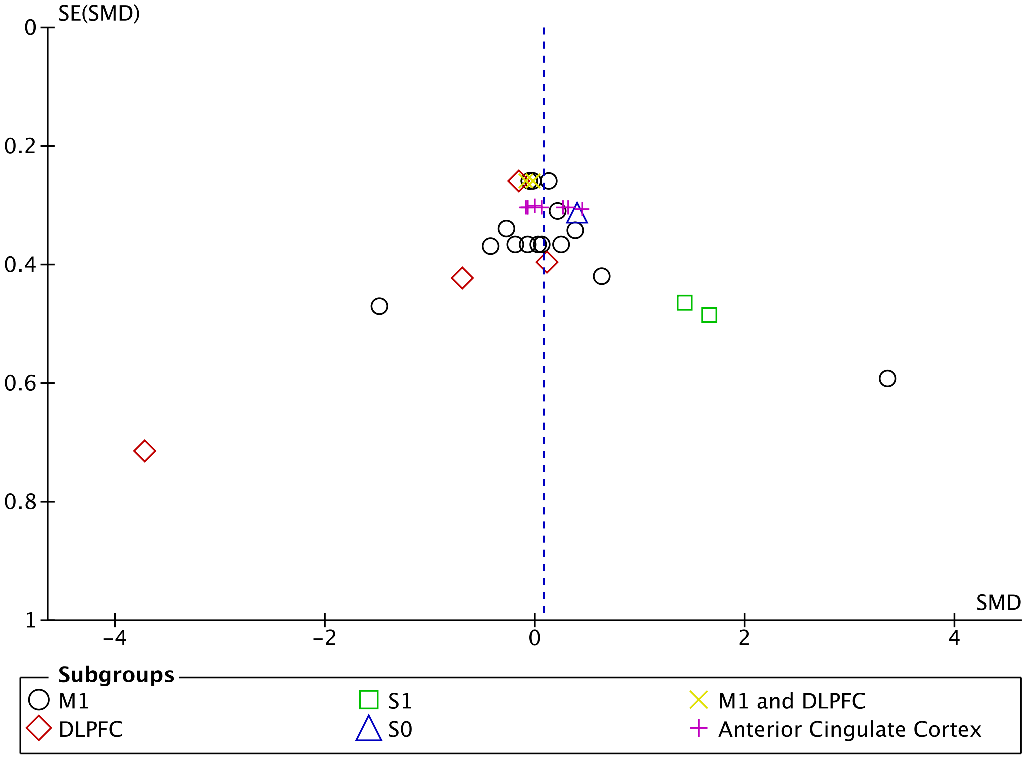

Figure S2

S4. Effects of tDCS on HPTs funnel plot.

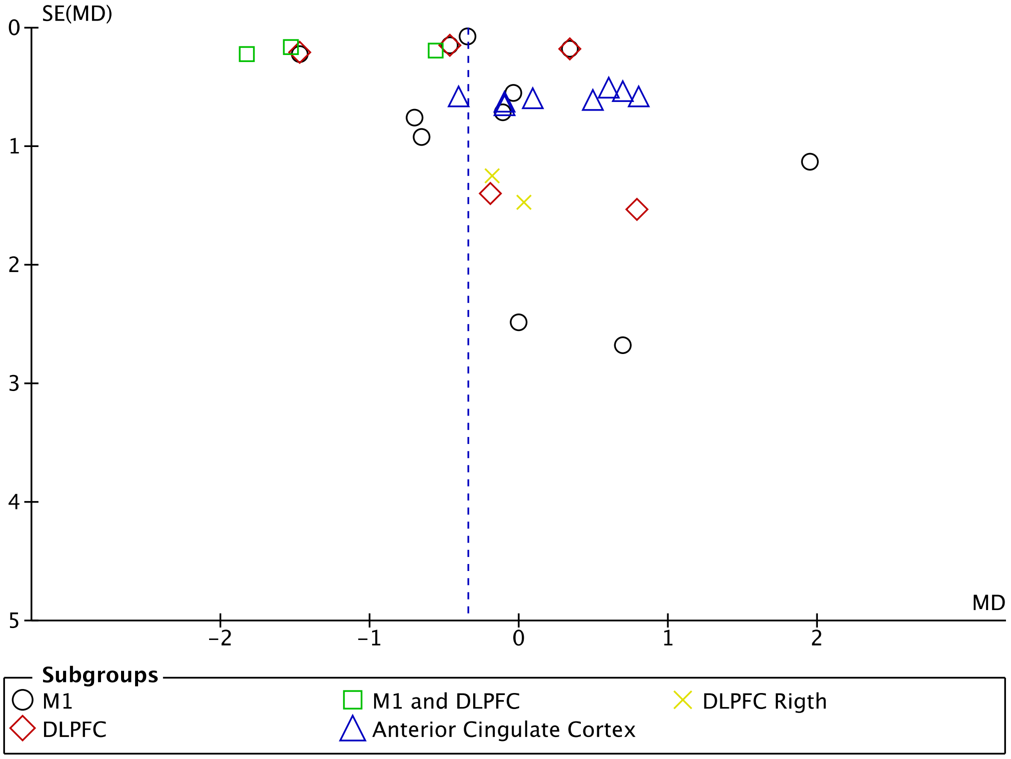

Figure S3

S5. Effects of tDCS on CPTs funnel plot.

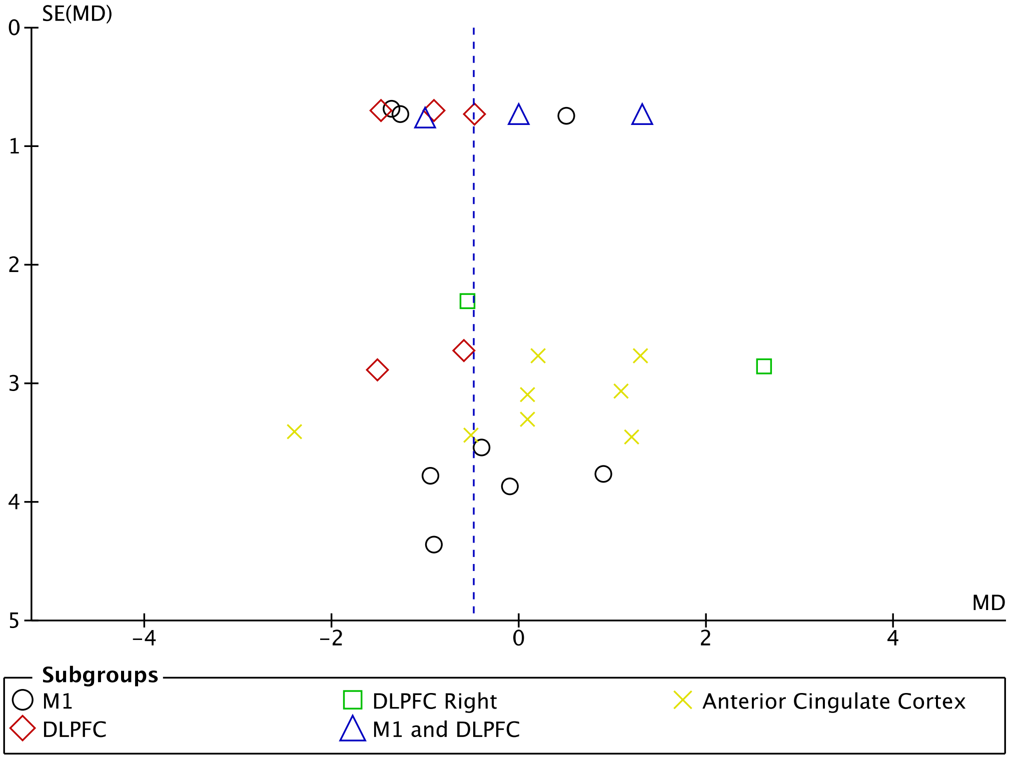

Figure S4

S6. Effects of tDCS on pain funnel plot.

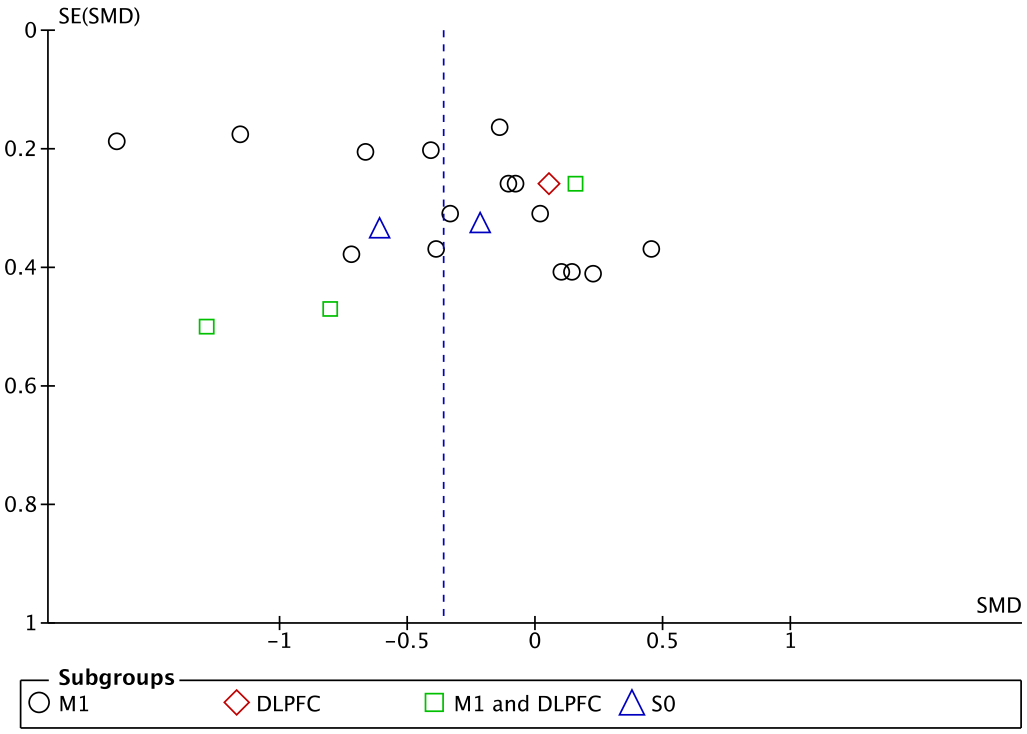

Figure S5
